# Chronic Pleuritis

**Published:** 1857-11

**Authors:** Addison Niles

**Affiliations:** Lockport, New York


ART. III.—*Chronic Pleuritis.* Cases reported by ADDISON NILES, M. D., Lockport, New York.

CASE I. March 27, 1857, visited James Trowley, æt. 13, native of England. From his mother I learned the following facts:

In infancy he was afflicted with a cutaneous eruption on the scalp, attended by an exfoliation of bone, having a depression still observed. The cure of the eruption was followed by a chronic diarrhœa, and discharges from the ears; from this time he has been an invalid to a greater or less extent. During the nine years past he has had more or less cough, pain and soreness in the left side, which were increased by taking cold.

One year ago last May, he was confined to the bed about three weeks, with fever, cough, and pain in the left side, which has always been in one location, occupying a space of about six inches in breadth, commencing at the nipple and extending to the base of the chest. The cough, debility and pain continued through the summer. He has now been confined to the bed about five weeks; has cough, pain in the left side, difficulty of respiration, and diarrhœa. He is supported in bed with his chest considerably elevated. Respiration 40 in a minute; pulse 120; face and lips blanched; feet, ankles and integuments, on the left side of the chest, œdematous; stomach very irritable, vomiting easily provoked by food and medicine. The left side of the chest measures three-fourths of an inch more than the right. On percussion it is dull throughout the whole extent; the sound on the right side is clear. The heart pulsates considerably to the right of the sternum. No respiratory murmur can be heard on the left side, except for a small space near the spine. On the right the respiration is puerile, and râles are heard indicating bronchitis. The urine is scanty, specific gravity 1.010 and highly albuminous.

The history of the case, symptoms, and physical evidence, clearly indicate chronic pleuritis with effusion. In forming a prognosis, the question arises, are the kidneys permanently diseased? The strumous and anæmic appearance of the patient, his long-continued ill-health, the albuminous urine, effu sion into the cellular tissue, irritable stomach and diarrhœa, tend to establish the probability of Bright's disease.

On the contrary, no renal epithelium, casts or oil globules can be detected by a careful examination of the urine by the microscope. The long-continuance of the cough, and pain in the chest; the recent occurrence of symptoms indicating renal affection, justify the belief that the disease of the chest is the primary one, and that the albuminuria is the consequence of contamination of the blood, arising from the absorption of pus or other fluids from the chest. Taking this view of the case, the prognosis is favorable, notwithstanding the formidable appearance it presents. The operation of paracentesis thorasis was proposed, and agreed to by the patient and his parents.

March 28. Visited him with Dr. Stork, who kindly assisted in the operation. Anæsthesia having been induced by a mixture of equal parts sulphuric ether and cloroform, the integuments of the chest were drawn outward by an assistant, an incision made one inch and one-half in length over the centre of the eleventh rib, near the junction of the posterior third with the anterior two-thirds of the chest. The skin being dissected upward to the upper margin of the rib, a puncture was made with the point of a lancet, and a trocar and canula passed into the chest. The trocar having been withdrawn, about one quart of healthy pus passed away; some air now entering the canula was withdrawn, and the integument which was raised up, allowed to fall down and close the orifice. Adhesive straps were applied, leaving the edges of the wound apart so as to prevent adhesion, and an opiate administered.

29th. The patient rested well, and feels better to-day. The canula is again introduced, and one pint of pus discharged, and a tent inserted to keep the orifice open. Thirty drops of tincture of chloride of iron is prescribed to be taken thrice a-day after meals; and one tablespoonful of the following mixture, thrice a-day, two hours after meals, and an anodyne at night:

℞ Olium morhuæ, ℥jv.
Olium carni, ʒj.
Vitellium oir, j.
Pulv. sachari albi, ℥ij. Mix.

The four successive days, the tent was withdrawn, and about eight ounces of pus passed each time.

April 4. The tent is removed, and a gum-elastic tube adapted to a stomach pump introduced, and one quart of pus pumped out.

5th. Eight ounces removed in the same manner.

6th. The tent is withdrawn and two ounces passed from the orifice.

From this time, for ten days, the tent was daily removed, and on an average four ounces of pus passed away.

14th. Symptoms much improved; appetite good; the œdema removed, but complains of pain in the right side.

Continue the oil and iron, and direct that the tent be daily removed and the matter be withdrawn by a catheter.

26th. Patient worse; no matter has passed for four days; difficulty of breathing increased and appetite wanting.

Introduced the tube, and by the pump drew out one pint of fœted pus.

28th. Patient better, although complaining of pain in the side.

29th. The catheter was again introduced, and about six ounces of bloody purulent fluid pumped out.

June 1st. Considerable pain was caused by the last operation. From this time the mother was directed to withdraw the tent daily, and allow the matter to flow through the elastic catheter.

Aug. 1st. The orifice has been closed about one week; about ten days previous to its closure the discharge was serous. The urine is now free from albumen, and the heart pulsates on its normal situation, and the vascular murmur is heard through the whole extent of the chest. The left side measures one-fourth of an inch less than the right. The chest is considerably flattened from the nipple downward to the base of the chest. The appetite, general health, and strength, are quite restored. A slight cough and expectoration still continues in the morning, and râles occasionally heard on the right ride, indicating some degree of bronchitis.

The patient advised to continue the cod liver oil.

Case II. July 12, 1842, performed the operation of thoracentesis on a son of John Connor, of Howard, Steuben Co. The patient, a boy of about 8 or 10 years of age, who had previously been under my care with an attack of accute pleuritis, which passed into a chronic stage. The disease affected the left side, which was considerably enlarged, and the heart was pushed to the right of the sternum. The operation was performed as described in case first, and about three pints of pus passed from the orifice; a tent was then inserted, and afterward daily withdrawn, to allow the matter to pass out. The treatment was tonic and alterative, and the patient, after a protracted illness, perfectly recovered, and was well when last heard from.

Case III. March 3, 1849 Operated for paracentesis thorasis, on a son of Thomas Morrow, of Bash, Steuben Co., 8 or 9 years old. This patient was affected when I first saw him, with chronic pluritis, of the left side, which was visibly enlarged, the heart pulsating to the right of the sternum. The operation was performed as in Case I, and about one quart of pus passed through the orifice; a tent was inserted and withdrawn at intervals of one, two three, four or seven days, and an elastic tube adapted to a stomach pump introduced, and the matter withdrawn by the pump. When the matter was suffered to remain as long as seven days, the patient was much worse; but was always decidedly relieved by pumping out the fluid. No memorandum having been made at the time in this case and Case II, the treatment can only be given from memory. Cod liver oil was the most important remedial agent administered. This was continued until his health was restored, since which time he has continued well, as far as I have heard.

Case IV. April, 1857, visited J. D., of Italy Hill, a girl about 10 years of age, in connection with Dr. Elisha Doubleday, of Italy Hill, and the late Dr. Wisner, of Penn Yan, both eminent practitioners. The patient was suffering from chronic pleuritis, with effusion; and having at times febrile symptoms. The operation was performed by Dr. Wisner, by puncturing the chest with a lancet; a large quantity of pus passed away, and a tent was introduced and subsequently withdrawn from time to time, in order to discharge the matter. The patient recovered for the time, but afterward removing from the place her subsequent history is unknown.

Case V. In the summer of 1848, visited G. D., aged about 23, in connection with the attending physicians, Drs. E. Doubleday and Wixom.

This case exciting more than common interest, an unusual number of practitioners were called in consultation. Among those were Dr. A. B. Case, of Howard, the late Dr. Oliver, of Penn Yan, and Dr. Rush Spencer, of Geneva. This case of pleuritis, although chronic in its nature, presented febrile symptoms occasionally. Thoracentesis was performed in my absence by the physicians in attendance; about two quarts of fluid, &c., discharged, which on cooling became gelatinous.

This gentleman fully recovered, and, I believe, continues well.

Case VI. July 24, 1850, performed the operation of paracentesis thorasis, at my office, in Bath, on John A. Van Wie, of Howard, Steuben Co., aged 25 or 30 years, who was suffering from chronic pleuritis, and drew off one quart of pus by the stomach pump adapted to a flexible tube. On the following day he returned and I attempted again to introduce the catheter, but the orifice being closed it caused considerable pain, and the patient left before the operation was completed, and did not again return. No further attempt was made to evacuate the chest, so far as I know. He died some months after.

*Remarks.* The above cases of chronic pleuritis complete the list of patients on which the operation of thoracentesis was performed by myself, and others under my observation. Judging from my limited experience, I should say the operation may be performed in every case of chronic pleuritis with a good prospect of success, where the disease is not complicated with some more grave disorder; and that the operation should be performed early, as soon as it is manifest that the effusion is purulent; or if serous that the fluid cannot be safely left for absorption. The early removal of the effusion brings the two surfaces of the pleura in contact, and favors a cure by adhesion, besides permitting the lung to expand, and preventing albuminuria which may occur from absorption of the fluid.

CASE VII. *Chronic Pleuritis of the left side terminating in death by Pleuro-pneumonia on the right.*

E. W. Lewis, a prominent and much respected merchant of this town, in the 52d year of his age, was attacked with pleuro-pneumonia the latter part of April last. The acute attack commenced by a chill and fever, followed with a stitch in the left side, and a short suppressed cough and difficulty of respiration. This patient had been in bad health since some time in August, 1856, when he was attacked by a nervous difficulty in the left side. He complained of rn unnatural sensation in the upper and lower extremities, and at times in the whole side. He frequently spoke of a sensation of pressure and pinching in the left side, and sometimes a feeling as though the skin and cords were too tense. He had several attacks of neuralgia in the left side of the face and head. These symptoms existed in connection with disorder of the digestive organs. About three weeks before this attack, a short cough and some difficulty of breathing occurred; the latter was aggravated in paroxysms. The pneumonia was latent in its character, and could be detected by physical evidence only. During this attack he could not rest comfortably in a recumbent position; this, together with difficult respiration, and a short cough continued, although considerably relieved at times, to the termination of life. The acute inflammation subsided in about two weeks, under the ordinary treatment. The appetite returned, and other evidences of convalescence.

Shortly after this time the left side of the chest became perceptibly enlarged, and on measurement was found one-fourth of an inch larger than the right, on which the respiration was purile, and the sound on percussion clear, while it was quite dull in the inferior part of the left. The difficulty of breathing now became very distressing, especially at night, accompanied with belching of gas from the stomach. The pulse was increased in frequency especially in the after part of the day, numbering 100 in a minute. It now became quite evident that although the disease of the lung had subsided, the inflammation of the pleura had become chronic.

These symptoms were decidedly relieved by the operation of a hydrogogue cathartic, and by counter-irritants, alterative diuretic remedies; but the relief was not permanent; in the mean time the appetite was good, and only temporarily impaired by cathartic remedies. These symptoms continued, though in a somewhat mitigated form up to the 26th of June, when the case passed from my-observation.

Shortly afterward a slight diarrhœa occurred, and the belching of wind subsided; but the other symptoms, with a gradual failure of strength, continued. In the last weeks of his life albuminuria occurred, with œdema in the entire lower part of the body and left arm. About two weeks preceding death the appetite failed, and during the last week he expectorated rusty colored matter, complained greatly of want of breath.

August 24th. The body was examined, which was considerably emaciated; the left side of the chest measured one-fourth of an inch less than the right. The lower portion of the body, with the lower extremities, were œdematous. On opening the chest the right side was found to contain several pints of serous fluid, which appeared to be made up of serum and some blood, with flakes of coagulated lymph. The fluid was situated in the inferior part of the chest, pushing the lung upward and inward. The diaphramatic and the inferior portion of the costal pleura of the right side. was intensely red and rough, and portions of it covered with deposits of lymph still soft.

The right lung exhibited the effects of inflammation; the lower portion of the inferior lobe was hepatized. The pleura of the left side was red and injected, and a few ounces of clear serum found in the cavity. The left lung was free from disease. The kidneys were examined, but no morbid appearance was observed. The abdominal viscera appeared healthy.

*Remarks.* Chronic pleurisy is often mistaken for dropsy of the chest, and a correct diagnosis is of great importance, not only in its therapeutic relations, but likewise in deciding the question of thoracentesis, which is rarely called for in the latter disease. According to the late as well as the older authorities, the two diseases are not only individually distinct, but belong to an entirely different class of disorders: pleuritis to inflammation, hydrothorax to dropsy.

Dr. Flint, in his valuable work on the respiratory organs, says: "Serous effusion within the pleura not due to inflammation, constitutes the affection called hydrothorax. The effusion is purely serous, i. e. serum unmixed with inflammatory products." This definition is sustained by Laennec, Stokes, Dunglison, and Watson, and every authority who has written specially on diseases of the chest which I now have at hand to refer to.

Dr. Flint says, "it is never primitive or idiopathic. It always occurs as an effect or complication of some other disease, and in the great majority of cases coëxists with structural lesions of the heart and kidney."

Dr. Stokes says, he "has never seen a case of idiopathic hydrothorax." With Dr. Flint's clear definition before him, it seems scarcely necessary for the most inexperienced practitioner to err on this point.

Dr. Copland says: "Although pleurisy may terminate in and give rise to effusions in many instances, yet the grade of organic action is not the same in both," i. e. pleuritis and hydrothorax.

Dr. Wood states "that cases considered as idiopathic hydrothorax, are probably, in most instances, cases of high inflammatory irritation of the membranc, which has not yet reached the point of *inflammation*, simply because the vessels have relieved themselves by effusion."

Dr. Gross, in writing of acute pleuritis, says: "The quantity of the fluid poured out in this affection, varies from a few drachms to several quarts." He likewise states that "in a patient, fifty-two years of age, suffering from chronic pleuritis, he drew off full two gallons," while the quantity found in dropsy of the chest is usually small. It is quite unnecessary to assume the instance of the latter disease to account for effusion, in any case of inflammation of the pleura. To suppose that idiopathic dropsy of the chest could coëxist with pleurisy, would be absurd, because it presumes the existence of two different kinds of organic action, at the same time, and in the same place. Most of the errors in diagnosis on this point, have probably arisen from admitting the idea of idiopathic hydrothorax. Rejecting this imaginary form of the disease, there is little danger of mistake. Before the existence of dropsy of the chest can be admitted, it must ordinarily appear that a general dropsical diathesis, or some organic disease of the heart, kidney, or some mechanical obstruction to the circulation existed previous to its occurrence. So far as I have observed, some degree of febrile action and pain have occurred in every case of chronic pleurisy, while in dropsy of the chest they exist only as a complication, or depend on the organic cause which gives rise to it.

The prognosis in chronic pleuritis is ordinarily favorable; but not so with hydrothorax, because it is in most cases the consequence of some incurable organic disease.

